# A Case of Acute Tuberculous Pleuropneumonia in a Patient with Acute Lymphoblastic Leukemia

**DOI:** 10.1100/tsw.2010.52

**Published:** 2010-04-01

**Authors:** Snezana Zivanovic, Ljiljana Saranac, Gordana Kostic, Vesna Bogicevic, Danijela Jovancic

**Affiliations:** Pediatric Clinic, University Clinical Centre, Nis, Serbia

**Keywords:** tuberculosis, acute lymphoblastic leukemia

## Abstract

Respiratory system infections are the most common complications in immunocompromised cancer patients. We here report a 14-year-old male who was admitted to the hospital because of acute pneumonia, who had been diagnosed with acute lymphoblastic leukemia (ALL) when he was 12 years old. A diagnosis of acute tuberculous pleuropneumonia was made based on clinical and radiographical findings, and *Mycobacterium tuberculosis* was identified by Ziehl-Neelson acid-fast stain and culture on Löwenstein-Jensen medium. Twenty months before pneumonia onset, the patient had been treated with immunosupressive therapy (ALL IC-BFM 2002 protocol).

## INTRODUCTION

Infections are the main cause of pulmonary infiltrates in patients with hematological malignancies. Bacterial, fungal, and mycobacterium tubercular organisms are the main isolates. The host is immunocompromised by both malignancy and cytotoxic treatment. Mucosal destruction, humoral and cellular immunodeficiency, secondary to primary disease itself, and chemotherapy lessen the clearance of aspirated microorganisms and facilitate the local infection. There are some reports in the literature about patients with malignancy who developed tuberculosis (TB) during or after intensive chemotherapy[1,2]. In the present study, we report a case of acute tuberculous pleuropneumonia confined to the lower lung field in a patient with acute lymphoblastic leukemia (ALL) during the maintenance treatment (daily dose of Purinethol® [mercaptopurine] and methotrexate every week).

## CASE REPORT

A 14-year-old male was admitted to the hospital with fever, sweating, cough, and general fatigue for a month. A month before that, the patient visited a local hospital, where he received antibiotic treatment (ceftriaxone, cloxacillin) for pneumonia. However, both his condition and the chest radiography were aggravated in spite of the treatment. Subsequently, he was referred to the Pediatric Clinic (Nis, Serbia) for further evaluation. The diagnosis of leukemia was based on blood and bone marrow examinations in April 2006, and the patient was given treatment for the T-ALL high-risk therapeutic group (ALL IC-BFM 2002). At admission, he was in the maintenance phase of the therapy (daily dose of mercaptopurine and methotrexate once a week). Twenty months before TB set in, the patient was treated for ALL at the Hematology Ward. Initial clinical symptoms at admission were not conclusive: high body temperature, loss of appetite, and weariness. Clinical examination was remarkable for hepatomegaly, splenomegaly, and enlarged lymph nodes in the neck. In the peripheral blood work, the number of leukocytes was 89,900, hemoglobin concentration 78 g/dl, and thrombocytes 187 x 10-3μL. There were 95% of pathological blasts in the peripheral smear. Lactate dehydrogenase values were very high, 8912 mU/ml. Bone marrow examination showed 89% of lymphoblasts, type L1 and L2. Immunophenotypically, these cells originated in T-lymphocytic cell lines (T-I ALL). The bone marrow cell karyotype was 46xy. The cerebrospinal liquor examination was normal. The patient was treated according to the ALL IC BFM-2002 protocol, intermediary risk. On the eighth day, the patient had poor response to the treatment. He had more than 1000 blasts, which categorized him in the high-risk group. Thus, he continued receiving high-risk leukemia treatment according to Protocol I. The consolidation and reinduction phase showed no significant complications, unlike the maintenance phase in which, while taking a daily dose of mercaptopurine and methotrexate once a weak, he developed TB.

There was no history of TB in the family. The parent confirmed that he had been vaccinated with Bacillus Calmette-Guérin (BCG) 3 days after birth.

At admission, the patient's weight was 40 kg (P 10); his height 156 cm (P 10-25); his BMI 16.4 (P 9); his vital signs except body temperature 38.5°C; respiration rate, heart rate, and blood pressure were normal. The chest examination revealed decreased breath sounds in the left middle and lower lung fields. This area was dull on percussion. The BCG vaccine scar was visible (>3 mm).

Abnormal laboratory findings on admission were an increased sedimentation rate, C-reactive protein, neutrophilia, and lymphopenia with a total lymphocyte count of 1100 cells/mm$^3$ (Table 1). The absolute neutrophil count was high at 5900 cells/mm$^3$. The levels of serum electrolytes, blood urea nitrogen, creatinine, aspartate aminotransferase, alanine aminotransferase, and creatine phosphokinase were normal. Urinalysis revealed no hematuria or proteinuria. Blood and sputum cultures were negative. Ultrasonography showed splenomegaly. Bone marrow examinations showed hypercellular bone marrow, all three branches present at all developmental stages. No sign of blast. However, his infectious symptoms deteriorated and the consolidation on chest X-ray film expanded into the left lower lobe as reported by the radiologist (Figs. 1 and 2). Computed tomographic (CT) scan showed a high-density area occupying the left lower lobe with left pleural effusia (Fig. 3). Skin testing with five units of purified protein derivative of tuberculin produced no induration. Acid-fast bacilli (AFB) were present on the sputum smear stained with Ziehl-Neelsen when carried out three times. *Mycobacterium tuberculosis* was grown on Lowenstein-Jensen medium, and it was identified according to morphologic and biochemical properties of the isolate. Culture of the specimen obtained from sputum showed 50 colonies of *M. tuberculosis* at the sixth week. It was not drug resistant *in vitro.*

Treatment with empiric antibiotics (cefotaxime, vancomycin) was discontinued. Treatment with anti-TB drugs isoniazid, rifampicin, pyrazinamide, and ethambutol was initiated for 2 months according to the protocol for the initial phase of the TB treatment. The patient was also treated with prednisone for a month, where the initial dose was 40 mg and then was reduced by 5 mg each week until it was completely withdrawn. During the second week of illness, the patient became afebrile, while the parameters of inflammation normalized by the end of the second month of treatment. Radiographic results showed regression of changes and the resolution of the left-sided effusia. However, at the end of the second month, one sputum sample was AFB positive, while two other samples were negative. This was the reason to prolong the initial phase of treatment with the four antituberculotics for a month. At the end of the third month of treatment with the anti-TB drugs, all sputum samples were negative by AFB and the treatment was continued with two antituberculotics (isoniazid, rifampicin) for five more months. The total anti-TB treatment lasted for 8 months (3 + 5 = initial phase + observance phase) (Fig. 4). The treatment of leukemia was not disrupted, but was continued with methotrexate and mercaptopurine. It was finished in

**TABLE 1**
**Laboratory Findings on Admission**

| | |
|---|---|
| Hematology | |
| Red blood cells (/mm³) | $415 \times 10^4$ |
| Hemoglobin (g/dl) | 13.3 |
| Hematocrit (%) | 40.2 |
| Platelets (/mm³) | $19.5 \times 10^4$ |
| White blood cells (/mm³) | 7300 |
| Neutrophils (%) | 81.2 |
| Eosinophils (%) | 0 |
| Monocytes (%) | 4.1 |
| Basophils (%) | 0 |
| Lymphocytes (%) | 14.7 |
| Biochemistry | |
| Total protein (g/dl) | 6.1 |
| Albumin (g/dl) | 4.0 |
| Total bilirubin (mg/dl) | 0 |
| Glutamic oxalacetic transaminase (mU/ml) | 17.5 |
| Glutamic pyruvic transaminase (mU/ml) | 17.0 |
| Lactate dehydrogenase (mU/ml) | 605 |
| Creatine phosphokinase | 28.1 |
| Blood urea nitrogen (mg/dl) | 6.89 |
| Creatinine (mg/dl) | 0.4 |
| Na (mEq/l) | 143.1 |
| K (mEq/l) | 3.86 |
| Cl (mEq/l) | 101 |
| Ca (mEq/l) | 2.17 |
| P (mEq/l) | 1.4 |
| Parameters of inflammation | |
| Erythrocyte sedimentation rate (mm/h) | 20 |
| C-reactive protein (mg/dl) | 131 |
| Fibrinogen | 8.2 |
| Blood gas analysis (room air) | |
| pH | 7.44 |
| $P_kO_2$ (mmHg) | 75 |
| $P_kCO_2$ (mmHg) | 38 |
| Sputum | |
| Bacteriological and fungal culture | Negative |
| Sputum smear microscopy AFB | Positive |
| Mycobacterial culture | Positive |

line with the leukemia protocol, on the sixth month of TB treatment. During the treatment, the patient did not have any undesired side effects due to the anti-TB drugs or any relapses. An ophthalmological examination was normal. Transaminase, urea, creatinine, and acidum uricum were within normal values during the treatment, including both the initial and the observance phase. There were no hematologic abnormalities, e.g., anemia (undesired side effects on pyrazinamide), nor allergic reactions. After the TB

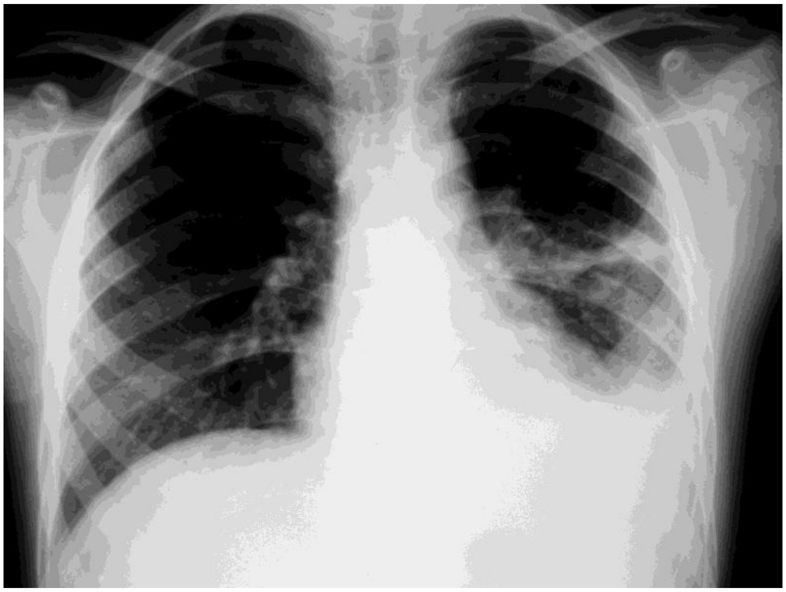

**FIGURE 1.** Lung radiography in PA position at the beginning of respiratory symptoms and 1 month before admittance to the Pediatric Clinic.

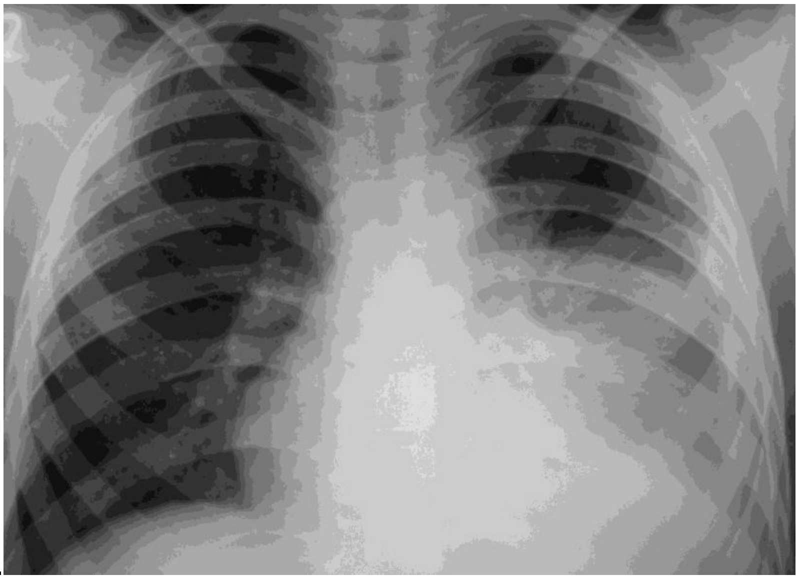

**FIGURE 2.** Lung radiography after admittance shows extensive parenchymal infiltrate of the lower left lobe of the left side (lung) with pleural effusion.

treatment, the patient was monitored for a year and was without respiratory symptoms, TB relapse, or relapse in leukemia.

## DISCUSSION

Although TB in Europe makes up a small percentage of the disease globally, its epidemiology reflects worldwide trends. There are major disparities between the rates of disease between resource-poor Eastern

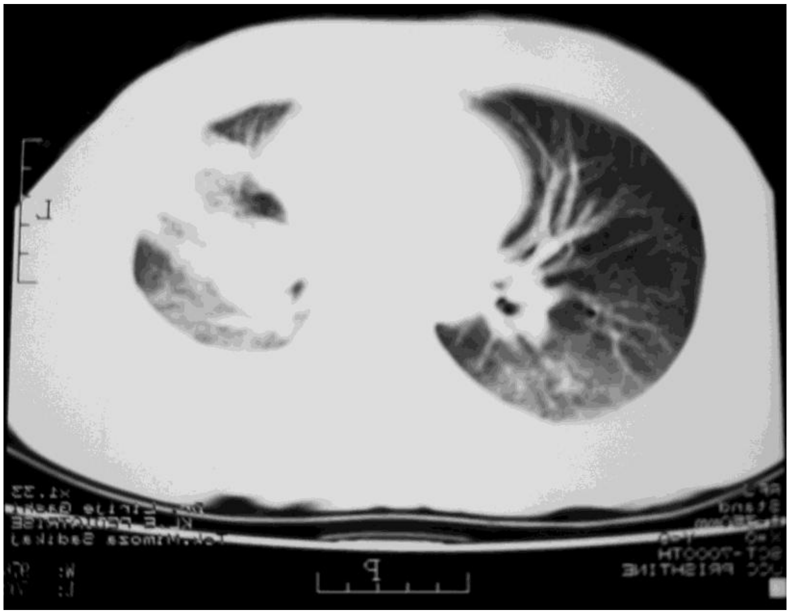

**FIGURE 3.** Two weeks before admission, a CT chest scan (taken at the beginning of respiratory problems, while in another local hospital), shows the area of high density in the lower left lobe with pleural effusia.

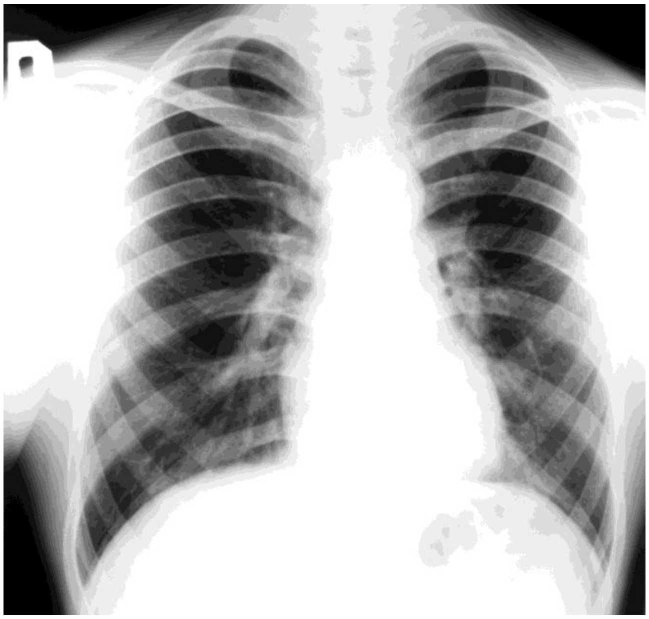

**FIGURE 4.** Lung radiography after the anti-TB drug treatment was over (at the end of the eighth month of treatment) shows a complete recovery.

countries and Western countries, which have the resources to fund TB control programs. Countries in Eastern Europe are seeing a resurgence of TB in local-born individuals related to multiple factors, including breakdown of TB control, multidrug-resistant TB, and HIV infections[3]. An increased risk for TB has been associated with different malignancies, including Hodgkin's and non-Hodgkin's lymphoma, lung cancer, head and neck tumors, hematological malignancies, as well as those following allogeneic bone marrow transplant[4]. Adzic reported on 20 adult patients with pulmonary TB and hematological malignancies of whom 40% were non-Hodgkin's lymphoma, 25% chronic lymphocytic leukemia, and 2% Hodgkin's disease[5]. Not many pediatric cancer patients are reported with pulmonary TB[6,7,8,9,10].

Hospital bloodstream infections are important causes of morbidity and mortality, and in many cases, their frequency is increasing due to the use of more intensive myelosuppressive treatments, particularly in oncology units. In the immunocompromised host, the risk of infection increases with the duration of neutropenia and immune impairment. A defect in cell-mediated immunity (CMI) is the fundamental immune defect placing immunosuppressed patients at risk for TB, rather than a defect in either neutrophil number or function[11,12]. However, in the setting of childhood ALL complicated by unresolved pneumonia with persistent pulmonary infiltrate, *M. tuberculosis* should be considered, particularly if TB is found in the extended family microenvironment and/or is prevalent in the community. The most important means of transmission of TB is by inhalation of aerosols carrying *M. tuberculosis*.

The family of our patient resides in Kosovo, where the incidence of TB is nearly three times higher than in Serbia (24 in 100,000). Potential TB sources were not determined. Close relatives and family members are all healthy. They were clinically examined; lung radiography and AFB sputum were within normal values. The adolescent was treated for leukemia for 20 months and spent a lot of time in the hospital, but had no contact with TB patients. During this period, he also had no contact with his school friends. The parents are not familiar with another case of TB in their surroundings. In the neonatal period, he was given a BCG vaccine. His BCG scar was longer than 3 mm. We assume that this is the case of reactivation of the endogenous latent focus of TB.

Childhood TB usually presents with fever, cough, lymphadenopathy, and loss of appetite and weight. Atypical presentations are also reported in children and adults[13,14,15,16,17]. Clinical symptoms are much less specific among children who are malnourished, immunocompromised, or suffering from HIV or malaria. Tuberculous pleural effusion is the most common form of extrapulmonary TB. It develops when a subpleural TB focus ruptures into the pleural space and elicits a significant immune response. This can happen after the primary phase (up to 6 months postexposure) or during the secondary phase (endogenous reactivation). Clinically, it often manifests with cough, pleuritic chest pain, dyspnea, low-grade fever, and other nonspecific constitutional symptoms. Radiographically, it is generally unilateral, small to moderate in size, more frequent on the right side, and sometimes associated with parenchymal disease. The sputum can be positive for TB from 4% in isolated TB pleurisy to 50% in cases with extensive parenchymal infiltrates. Even if not treated, the clinical course is toward spontaneous resolution, with minimal pleural scaring. Steroids may hasten the pleural fluid resorption and resolution of clinical symptoms, although they do not seem to prevent scar formation[18]. Our patient's tuberculous pleurisy was associated with parenchymal disease.

The gold standard for establishing the diagnosis of TB in adults is sputum smear microscopy for AFB confirmed by microbacterial culture. Culture of gastric juice or bronchoalveolar lavage for TB bacteria is positive in less than 50% of the cases[19,20]. Diagnosis of TB is more difficult in young children and immunosuppressed patients as both populations are less likely to develop a positive tuberculin skin test (TST), and less likely to have positive sputum staining for AFB[8]. Our patient had a negative TST because of the immunosuppressive illnesses or severe infection.

The treatment of TB in the context of ongoing immunosuppression is challenging and the published guidelines are not available. The response to the treatment of TB does not differ significantly between immunosuppressed and immunocompetent adults[21]. Good clinical response to TB treatment was reported in adult patients with underlying hematological malignancies treated with isoniazid, rifampicin, pyrazinamide, and ethambutol for 2 months followed by isoniazid and rifampicin for an additional 4–10 months[22]. Corticosteroids are frequently used for the complications of primary disease, particularly meningeal TB, tuberculous pericarditis, miliary TB, tuberculous pleurisy, and severe bronchial obstruction. Patients with severe and persistent toxic symptoms can also benefit[23,24]. The response to the initial 2-month anti-TB regimen was partial. Despite the clinical improvement, radiographical regression, and normalization of inflammation parameters, one sputum sample was AFB positive at the end of the second month. AFB was negative in all three samples at the end of the third month of treatment. During the observation phase, the 5 months while taking dual anti-TB drugs, the patient had no undesired side effects or relapses.

## CONCLUSION

The prevention of polymicrobial infection in immunocompromised children is imperative due to its high morbidity and mortality. Oncologists need to target their empirical antibiotic therapy for the prevalent hospital-acquired infections, taking into consideration the antibiogram of the hospital isolates. However, unusual pathology such as TB must also be considered and should be included in the diagnostic workup of such immunocompromised children. Gastric aspirates or bronchoscopic lavage fluid should be obtained at early stages for microbial identification, because children cannot produce sputum easily. Further investigations are needed in order to optimize the diagnosis and management of TB in pediatric patients, especially those with underlying malignancies.
